# Interpreting behaviors from accelerometry: a method combining simplicity and objectivity

**DOI:** 10.1002/ece3.1660

**Published:** 2015-10-02

**Authors:** Philip M. Collins, Jonathan A. Green, Victoria Warwick‐Evans, Stephen Dodd, Peter J. A. Shaw, John P. Y. Arnould, Lewis G. Halsey

**Affiliations:** ^1^ School of Life SciencesUniversity of RoehamptonHolybourne AvenueLondonSW15 4JDUnited Kingdom; ^2^ School of Environmental SciencesUniversity of LiverpoolLiverpoolL69 3GPUnited Kingdom; ^3^ Royal Society for the Protection of BirdsNorth Wales OfficeBangorLL57 4FDUnited Kingdom; ^4^ School of Life and Environmental SciencesDeakin UniversityMelbourneVictoria3125Australia

**Keywords:** Accelerometer, behavior, data logger, human, kittiwake, objective, simple

## Abstract

Quantifying the behavior of motile, free‐ranging animals is difficult. The accelerometry technique offers a method for recording behaviors but interpretation of the data is not straightforward. To date, analysis of such data has either involved subjective, study‐specific assignments of behavior to acceleration data or the use of complex analyses based on machine learning. Here, we present a method for automatically classifying acceleration data to represent discrete, coarse‐scale behaviors. The method centers on examining the shape of histograms of basic metrics readily derived from acceleration data to objectively determine threshold values by which to separate behaviors. Through application of this method to data collected on two distinct species with greatly differing behavioral repertoires, kittiwakes, and humans, the accuracy of this approach is demonstrated to be very high, comparable to that reported for other automated approaches already published. The method presented offers an alternative to existing methods as it uses biologically grounded arguments to distinguish behaviors, it is objective in determining values by which to separate these behaviors, and it is simple to implement, thus making it potentially widely applicable. The R script coding the method is provided.

## Introduction

Behavior is a manifestation of movement and can account for a large proportion of energy expenditure (Karasov 1992; Rezende et al. 2006), thus allocation of time to different behaviors can greatly affect an individual's survival and reproduction (Nagy et al. 1999). Behavior can be quantified over a range of biological scales, from within individual changes over short time‐scales (e.g., changes in behavior while foraging (Ropert‐Coudert et al. 2004)), to persistent changes in group behavior over time (e.g., changes in time‐spent foraging in response to increased interspecific competition (Namgail et al. 2006)). Yet, despite its importance, collecting sufficiently accurate, quantitative data on behavior for free‐ranging animals tends to be problematic, especially in motile and/or elusive species (Ropert‐Coudert and Wilson 2005). To address this, a range of biotelemetry approaches have been, and continue to be, developed to monitor animals remotely (Cooke et al. 2004). The most widely used biotelemetry devices collect positional data, and such devices have provided invaluable insights into species distributions across a range of spatial and temporal scales (Cagnacci et al. 2010). However, to elucidate behavior from such positional data alone is complex, typically involving either making assumptions (Freeman et al. 2010), introducing statistically complex behavior assignments (Guilford et al. 2009; Cristescu et al. 2014), or coupling the data with those obtained from other devices (Dean et al. 2013).

Among these other devices, the use of accelerometers to identify behaviors in free‐ranging animals has become increasingly common in recent years (Yoda et al. 2001; Tsuda et al. 2006; Halsey and White 2010; Zimmer et al. 2011; Williams et al. 2014). Accelerometers measure the acceleration of an organism across one, two, or three axes. By measuring across multiple axes, it is possible to derive the orientation of the logger which, in relation to gravitational force, in turn makes it possible to derive the orientation of the instrumented animal (Tsuda et al. 2006; Halsey and White 2010; McClune et al. 2014). The moment‐to‐moment difference between the acceleration recorded by the logger and the orientation of the logger indicates the dynamic movement of the animal's center of mass (Gleiss et al. 2011). Accelerometers confer the advantage over direct observations and inference from other biologging tools, such as GPS loggers, of being able to record at high temporal resolutions (from 0.5 to 10,000 Hz), allowing measurement of short‐lived behaviors such as escape responses or feeding events (Carroll et al. 2014; Kawabata et al. 2014) as well as continuous measurement of coarse‐scale behaviors such as flight, resting, swimming, and running (Shepard et al. 2008; Halsey et al. 2009; McClune et al. 2014).

However, identifying discrete behaviors in accelerometry data at all temporal scales has to date largely involved subjective assessments of data or, as with identifying behavior from positional data, the use of complex computational techniques; both of which often lack validation (Bidder et al. 2014). This lack of consistency has resulted in numerous techniques being developed for classification of such data. The simpler methods available in the literature tend to be reliant on separating behaviors by specific threshold values of metrics derived from acceleration data. These are typically determined through comparison with a source of validation such as video‐recorded images (Kawabata et al. 2014), or through subjective inspection of the data (Gómez Laich et al. 2009); in both cases such approaches are, therefore, largely study‐specific and potentially labor intensive. Furthermore, despite their efficacy, objectivity, and increasing availability in statistical software packages (Nathan et al. 2012; Campbell et al. 2013; Gerencsér et al. 2013; Bidder et al. 2014; Carroll et al. 2014), approaches based on machine learning, which are also reliant on a source of validation and comprise numerous types of analyses, are conceptually difficult and therefore potentially inaccessible to many biologists. Indeed, such complexities may discourage the collection and use of accelerometry data. A computationally simple method for interpreting behaviors from accelerometry data, which is not inherently reliant on a source of validation yet which also incorporates objectivity, is currently lacking. A key consideration which emerges when evaluating and choosing methods to interpret such data is the level of information required to answer the target research questions. In many studies, this might mean that just the coarse‐scale behaviors need to be identified; for example, when comparing time‐activity budgets between individuals or groups (Gómez Laich et al. 2011; Le Vaillant et al. 2012) or for isolating certain behaviors to calculate associated energetic costs (Wilson et al. 2006). Even for studies identifying finer‐scale behaviors and short‐lived events such as characteristics of limb movement during locomotion, identifying the coarse‐scale behaviors is often a necessary first step in analysis (Kawabata et al. 2014).

This study presents a computationally simple method for assigning coarse‐scale behaviors to accelerometry data. Discrete behaviors are assigned by using objectively identified separation points in frequency histograms of simply calculated metrics derived from accelerometry data. Behavioral assignments using this method are presented and independently validated for two distinct species with disparate modes of locomotion: black legged kittiwakes *Rissa tridactyla* and humans *Homo sapiens*.

## Materials and Methods

### Data collection

Tri‐axial accelerometers (X8m‐3; Gulf Coast Data Concepts, LLC, MS, USA; recording range ±8 g, resolution: 0.001 g, weight: 14 g), set to record at 25 Hz, were attached to feathers on the center of the backs of seven kittiwakes using clothed black Tesa^®^ tape. The placement of the accelerometer was kept as consistent as possible across all birds. In addition to the accelerometers, birds were deployed with salt water immersion loggers (GLS Mk18‐H British Antarctic Survey, weight: 1.9 g) on the tarsus via cable tie attachment to existing metal leg rings. These loggers record a value between 0 and 200 once every ten minutes, measuring the proportion of time the logger was immersed in salt water over the previous epoch. Average body mass was 357 ± 20 g (mean ± SD) and data loggers weighed on average 4.5 ± 0.2% of body mass, which is within recommendations for deployment weight (Bridge et al. 2011). All seven birds were recaptured but one of the salt water immersion loggers was not functioning upon removal, giving a final sample size of six combined deployments. Deployment time ranged from 47 to 74 h during which time birds exhibited normal breeding behavior, including incubation of eggs, rearing of chicks (dependent on which breeding stage they were at), or the absence from the nest (most likely on foraging trips). Fieldwork was carried out on Puffin Island, North Wales (53° 19′ 05″ N, 04 °01 ′40″ W) in July 2013. All work was carried out under Countryside Council for Wales permit number (44043:OTH:SB:2013).

The same tri‐axial accelerometers set to record at 25 Hz (*n* = 5) or 40 Hz (*n* = 1) were attached to the sternum in a vertical orientation using Tesa^®^ tape on six humans. Participants were instructed to undertake three activities for approximately five minutes each: sitting, walking, and running. All participants carried out each of the activities once and in the same order. Duration of deployment ranged from 14 to 28 min.

### Approach

**Figure 1:**
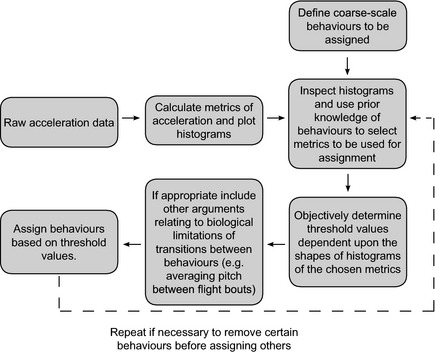
Flowchart of the process developed for assigning behaviors to accelerometry data.

The method of behavioral assignment presented here consists of a stepwise process which assigns predetermined behaviors to acceleration data using objectively identified threshold values of metrics derived from raw acceleration data (outlined in Fig. 1). Initially, behaviors to be classified were considered and metrics thought likely to differ depending on these behaviors were calculated from raw accelerometry data. Histograms of these metrics were then plotted to identify any patterns potentially indicative of discrete behaviors. These histograms, coupled with knowledge of the target species and the target behaviors, were then used to select the metrics most suitable for assigning behaviors from the accelerometry recordings. Behaviors were assigned dependent upon threshold values of these metrics. These thresholds were objectively determined values relating to the shape of the histograms, specifically the minimum frequency of data points falling between peaks (the interpeak frequency minimum).

### Calculating metrics of acceleration

To identify metrics potentially indicative of discrete behaviors in both kittiwakes and humans, the following 10 metrics were calculated to 1‐sec intervals across the dataset of each subject bird or participant: mean acceleration and standard deviation of raw acceleration for each of the three axes (heave, surge, and sway), pitch of the body, and roll of the body; ODBA (overall dynamic body acceleration); and VeDBA (vectorial dynamic body acceleration). Mean and standard deviation of the acceleration values were calculated over a moving period of 25 data points (representing a duration of 1‐sec). Pitch (the angle of the device and therefore also of the bird or participant) and roll (the side‐to‐side movement of the bird or participant) were derived from all three axes using the following equations:

$$\text{Pitch}=\text{Arctan}\left(X/{\left(Y^{2}+Z^{2}\right)}^{1/2}\right)*\left(180/\text{pi}\right)$$

$$\text{Roll}=\text{Arctan}\left(Y/{\left(X^{2}+Z^{2}\right)}^{1/2}\right)*\left(180/\text{pi}\right)$$

where *X* is acceleration (g) in the surge axis, *Y* is acceleration (g) in the sway axis, and *Z* is acceleration (g) in the heave axis.

Overall dynamic body acceleration and VeDBA are measures of DBA (dynamic body acceleration) in all three dimensions. DBA was calculated by smoothing data for each axis across a 1‐sec period to calculate the static acceleration, and then subtracting the static acceleration values from the raw acceleration values. ODBA is the sum of the dynamic body acceleration of the three axes, whereas VeDBA is the square root of the sum of the squares of dynamic body acceleration of the three axes (Qasem et al. 2012).

### Assigning behaviors

We aimed to categorize kittiwake behaviors as: flying, on land, and on water, while human behaviors were categorized as: sitting, walking, and running. Assignment of behaviors was undertaken in a stepwise manner for both kittiwakes and humans. Metrics of the recorded acceleration data were selected based on how clearly they appeared to distinguish these target behaviors. Then, one behavior at a time was separated from the others based on a threshold value calculated as an interpeak frequency minimum of the metric employed. For the kittiwake data, flight behavior was assigned first on the basis that this dynamic movement was likely to be more distinct than the stationary behaviors of “on land” or “on water.” The behaviors of “on land” or “on water” were then assigned to the remaining data. For human data, sitting was assigned before “walking” and “running” were assigned, again on the basis that this stationary behavior was likely to be more distinct than the behaviors relating to two types of movement, walking and running.

**Figure 2:**
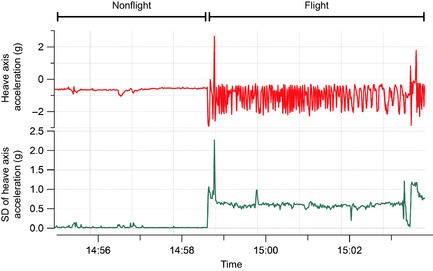
Raw acceleration values of the heave axis (upper trace) compared to the standard deviation of the heave axis (lower trace) from an accelerometer attached to a kittiwake.

Histograms plotted for the 10 metrics derived from the accelerometry data indicated that the standard deviation of the heave axis (SD_Heave_) was bimodal for all kittiwakes (Appendix S1) and trimodal for all humans (Appendix S2). SD_Heave_ also had the greatest range of values when compared to other axes, indicating that movement across this axis was the most variable. For these reasons, as well as the use of heave in previous studies to identify flight behavior (Wilson et al. 2006; Sato et al. 2008; Sakamoto et al. 2013; Vandenabeele et al. 2014), SD_Heave_ was the metric used to separate flight from nonflight behavior in kittiwakes, and to separate sitting, walking, and running in the human dataset. Furthermore, use of the standard deviation is likely to be more appropriate for identifying movement than just the raw acceleration values as raw acceleration during movement tends to oscillate and therefore likely overlap considerably with values recorded when the subject/participant is not moving (Fig. 2). As histograms of SD_Heave_ for kittiwake data were bimodal, it was expected that nonflight behavior would correspond to the lower values of SD_Heave_ and the higher values of SD_Heave_ would relate to flight. Therefore, the value of SD_Heave_ corresponding to the interpeak frequency minimum between the first and second peak was determined and used as the threshold value to separate these behaviors. Histograms of the human data had trimodal distributions of SD_Heave_ and, considering the three behaviors recorded in the data correspond to different amounts of movement, it was assumed that each peak related to each of the behaviors. As such, the SD_Heave_ value corresponding to the interpeak frequency minimum values between the first and second peak for each individual was determined and used as the threshold value for separating sitting behavior from walking and running. The value of SD_Heave_ corresponding to the interpeak frequency minimum between the second and third peak was determined and used as the threshold value to separate walking and running.

For kittiwakes, the behaviors of “on land” and “on water” were assigned after flight had been assigned. Therefore, histograms of calculated metrics were reassessed with data corresponding to flight removed (Appendix S3). Body pitch was chosen as the most suitable metric to use to separate these remaining behaviors. This is because a kittiwake's body angle is likely to be different when on land compared to on water, due to the influence of nest angle as well as differences in body position arising from the range of movements; notably standing, incubating eggs, and brooding chicks. Histograms of pitch showed clear peaks, indicating that individuals exhibited certain body pitch angles more predominantly than others during the data logger deployment (Appendix S3). The threshold value for separating “on land” and “on water” was determined as the pitch value corresponding to the minimum frequency value between the first and second peak in the pitch histogram for each bird.

**Figure 3:**
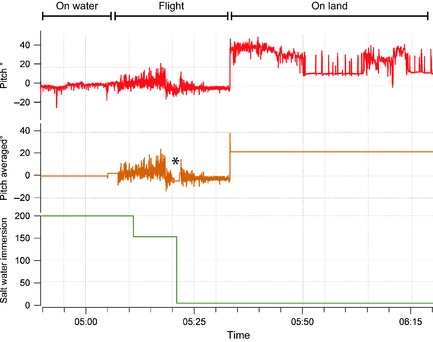
Pitch values of a kittiwake averaged to 1‐sec values (upper panel), and pitch values subsequent to the application of a correction factor averaging pitch between the end and start of flight periods (middle panel). Salt water immersion data, indicating on water or out of water (lower panel). The asterisk indicates a brief period of resting on water in the middle of the flight section.

Cliff‐nesting birds such as kittiwakes must fly to commute between land and water, thus to potentially further aid in the separation of the behaviors “on land” and “on water” this understanding of the underlying biology was incorporated into the behavioral assignment process. To prohibit the possibility of an assignment of “on water” directly following “on land” and vice versa without a period of flight in between, the mean pitch was calculated between the end of each bout of flight and the start of the next (Fig. 3). Data within the between flight bouts were then assigned as being “on land” or “on water” depending on the mean pitch value across the entire between‐flight period. These behaviors were assigned using the threshold determined by the interpeak frequency minimum from the histograms of pitch before averaging.

### Validation

To determine the suitability of assigning behaviors by thresholds that correspond to interpeak frequency minimum values of the chosen acceleration‐derived metrics, the accuracy of behavior assignments determined by a range of threshold values including the interpeak frequency minimum values was calculated. To determine accuracy, the behavioral assignments across these threshold ranges were calculated during periods when the behaviors were known. This validation step is not integral to assigning behaviors and was used in this instance to test the effectiveness of the presented method.

For kittiwakes, “flight” was assigned across a range of thresholds of SD_Heave_, from 0 to 1 *g* at 0.02 *g* intervals. “Flight” was assigned to data falling above each threshold. Accuracy of flight assignment dependent on the range of thresholds was calculated before assignment and subsequent validation of “on land” and “on water” behaviors. For assessing accuracy of assigning the behaviors “on land” and “on water” dependent on body pitch, the two behaviors were assigned across a range of pitch thresholds from −10° to 40° at 1° intervals. Data with pitch values below the threshold were assigned as “on water,” and data with pitch values above were assigned as “on land.” The intervals chosen for the range of thresholds (0.02 g for SD_Heave_ and 1° for pitch) correspond to the bin sizes used for plotting the histogram. Bin sizes chosen resulted in smooth histograms with sufficient resolution to detect small changes in posture or amount of movement. An examination of the effect of bin size across orders of magnitude indicated that it made almost no difference to the accuracy of behavioral assignment (Appendix S4).

The known period of behavior for kittiwake data used to calculate accuracy of assignment consisted of a two‐hour period for each bird encompassing the three target behaviors (flight, on land, and on water) which was selected by eye and was manually assigned behaviors as carried out previously with similar datasets (Bidder et al. 2014; McClune et al. 2014). Due to the varied time budgets of the individual birds, the amount of time within this two‐hour period spent doing each of the behaviors varied. Manual behavioral assignments were made using the programme IGOR Pro (Wavemetrics Inc., Portland, OR, USA, 2000, version 6.3.5) with the Ethographer package (Sakamoto et al. 2009). Flight was assigned when traces of acceleration data displayed periodic fluctuations in dorso‐ventral movement, as described previously in the literature (Wilson et al. 2006; Sato et al. 2008; Sakamoto et al. 2013; Vandenabeele et al. 2014), while assignment of “on land” or “on water” was informed by values from the salt water immersion logger. To calculate accuracy of assignment, we compared the assignment of behavior for every second for each threshold value to these known behaviors during the validation period. We were then able to calculate the percentage of behavioral assignments which were correct for each threshold value in the series.

For the human data, SD_Heave_ was used to assign all three behaviors. For assessing the accuracy of assigning sitting behavior, “sitting” was assigned to data with an SD_Heave_ value below a threshold between 0 and 2 *g* at 0.02 *g* intervals. Once sitting was assigned using the interpeak frequency minimum value of SD_Heave_, the behaviors of walking and running were assigned to the remaining data across a range of standard deviation thresholds. The thresholds ranged from the standard deviation value identified for separating sitting behavior (~0.1 *g*) up to a standard deviation value of 2.0 *g,* at 0.02 *g* intervals. As with the kittiwake data, intervals tested corresponded to the bin size of the histograms (0.02 *g*), with the chosen bin sizes resulting in smooth histograms. Furthermore, bin size again made very little difference to the accuracy of behavioral assignment (Appendix S4). Walking was assigned to data with a standard deviation below each threshold, while running was assigned to data above the threshold. Accuracy of human data assignments was easier to measure as during data collection exact activities were recorded by participants thus behavioral assignments were fully validated. Accuracy was calculated as the percentage of behavioral assignments from this method which were the same as the known, recorded behaviors. As deployments were relatively short the full dataset was compared to each threshold‐dependent assignment, giving a measure of accuracy across the full deployment.

All data analysis was conducted in R statistical software (R Development Core Team, 2015), other than visualization of accelerometry and immersion data for validation, which was conducted using the Ethographer package in Igor Pro (Wave Metrics). Script required to execute this method in R is provided (Appendix S5) along with an example dataset for a kittiwake (Appendix S6).

## Results

### Kittiwakes

**Figure 4:**
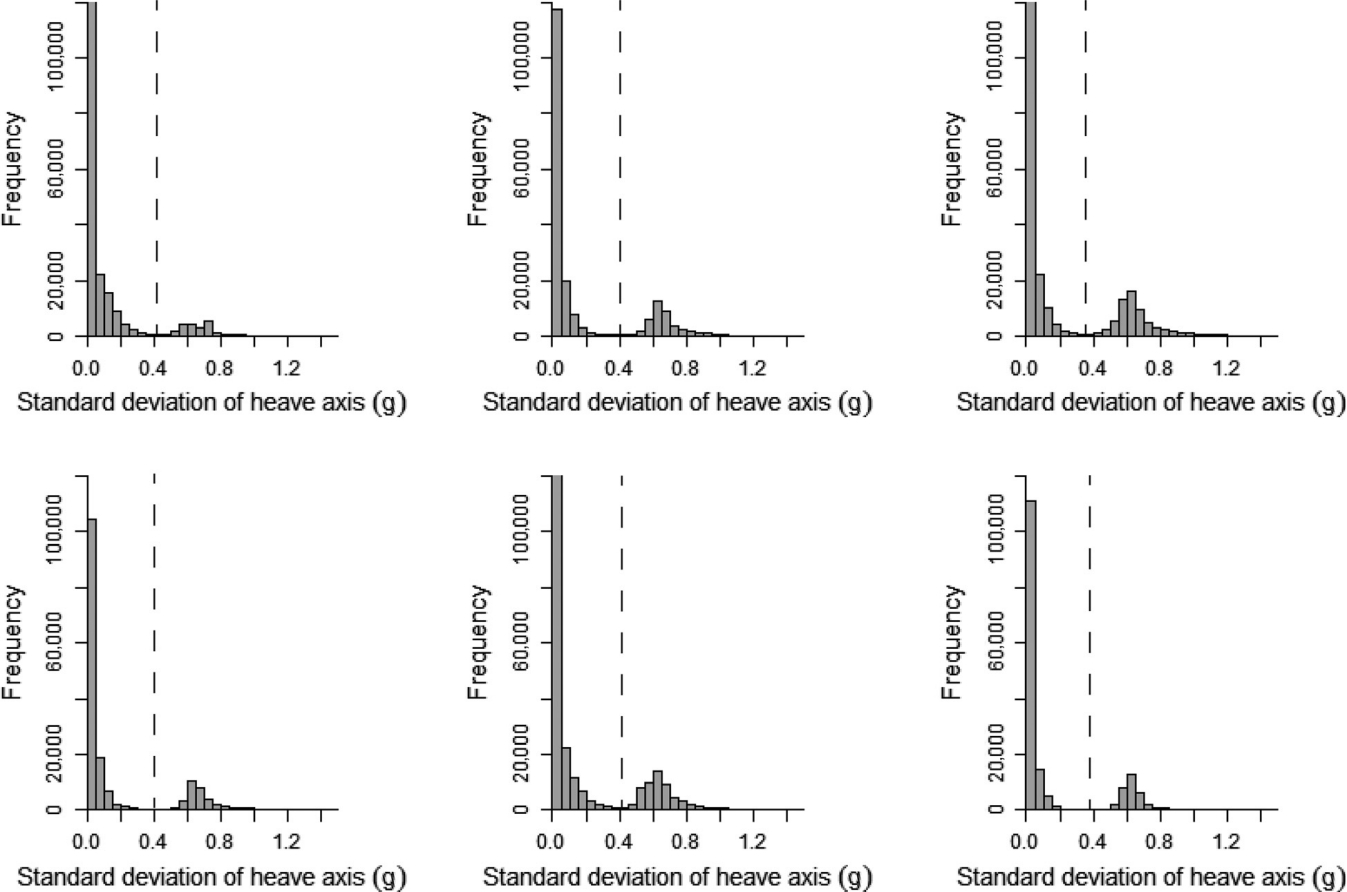
Histograms of the standard deviation of the heave axis data recorded during accelerometer deployments on each of six kittiwakes. The dashed line indicates the inter‐peak frequency minimum.

**Figure 5:**
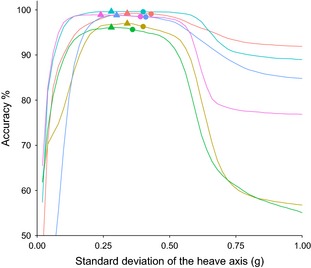
Percentage accuracy of flight assignment plotted against the standard deviation of the heave axis used as a threshold value used to assign the behavior. Each line represents an individual kittiwake. Circles indicate accuracy at the threshold value corresponding to the interpeak frequency minimum from the histogram of standard deviation of the heave axis (see Fig. 4), triangles indicate the value corresponding to the threshold value that achieves maximum accuracy.

A clear bimodal distribution was present in histograms of SD_Heave_ for all birds (Fig. 4). Separating flight behavior from nonflight behavior in kittiwakes using SD_Heave_ was highly accurate. By separating flight behavior using the interpeak frequency minimum threshold, the mean (±1SD) accuracy of assignment of flight versus nonflight behavior across all birds was 97.9 ± 1.7% (Fig. 5). Although this value did not correspond to the mean highest possible accuracy calculated across the full range of SD_Heave_ thresholds (98.3 ± 1.3%), the difference in accuracy was small (mean difference: 0.4 ± 0.3%; maximum difference: 0.9%).

**Figure 6:**
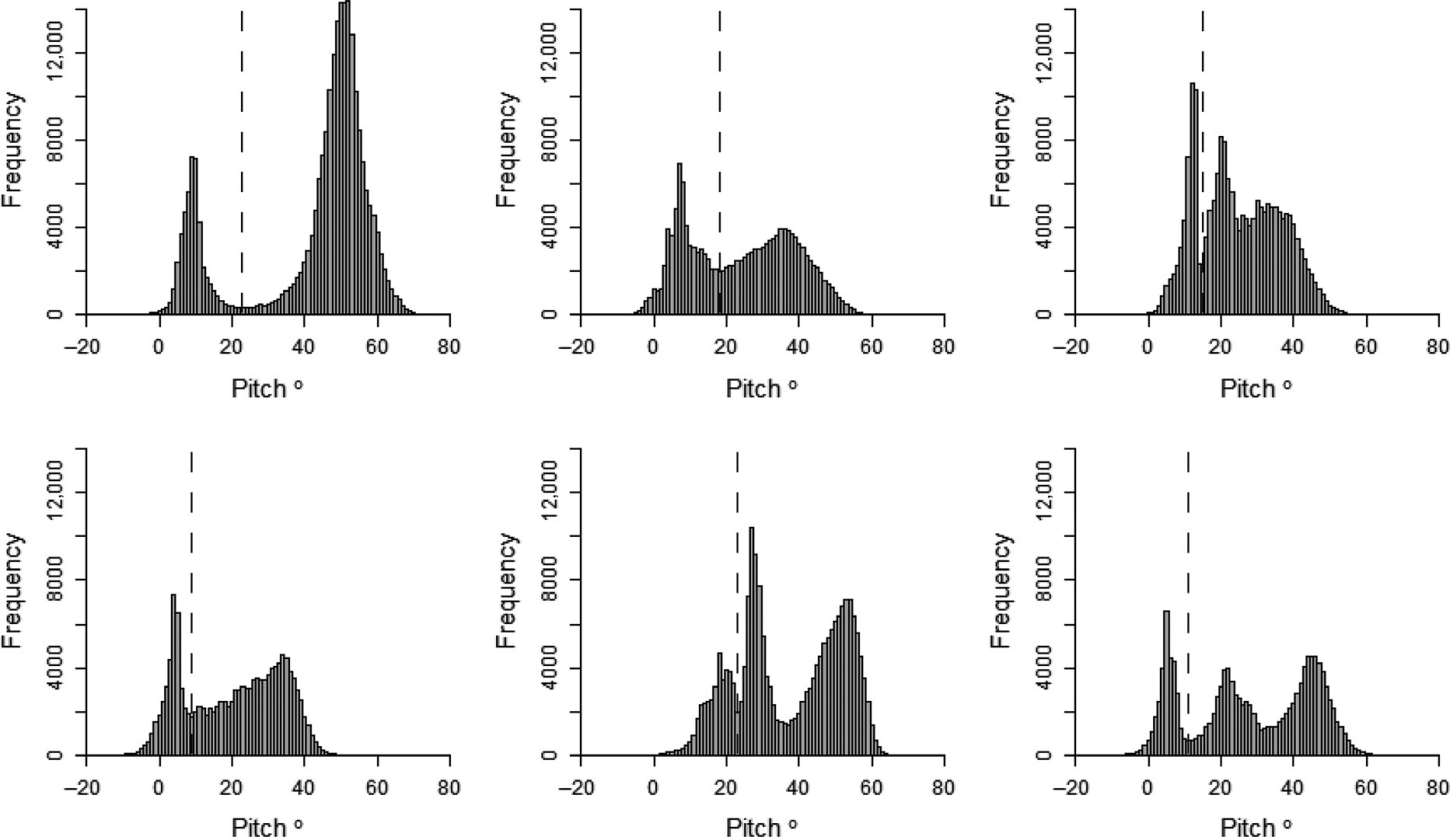
Histograms of the pitch angle of six kittiwakes while instrumented with an acceleration data logger. Data already assigned as flight are excluded. The dashed line indicates the interpeak frequency minimum between the first and second peak.

**Figure 7:**
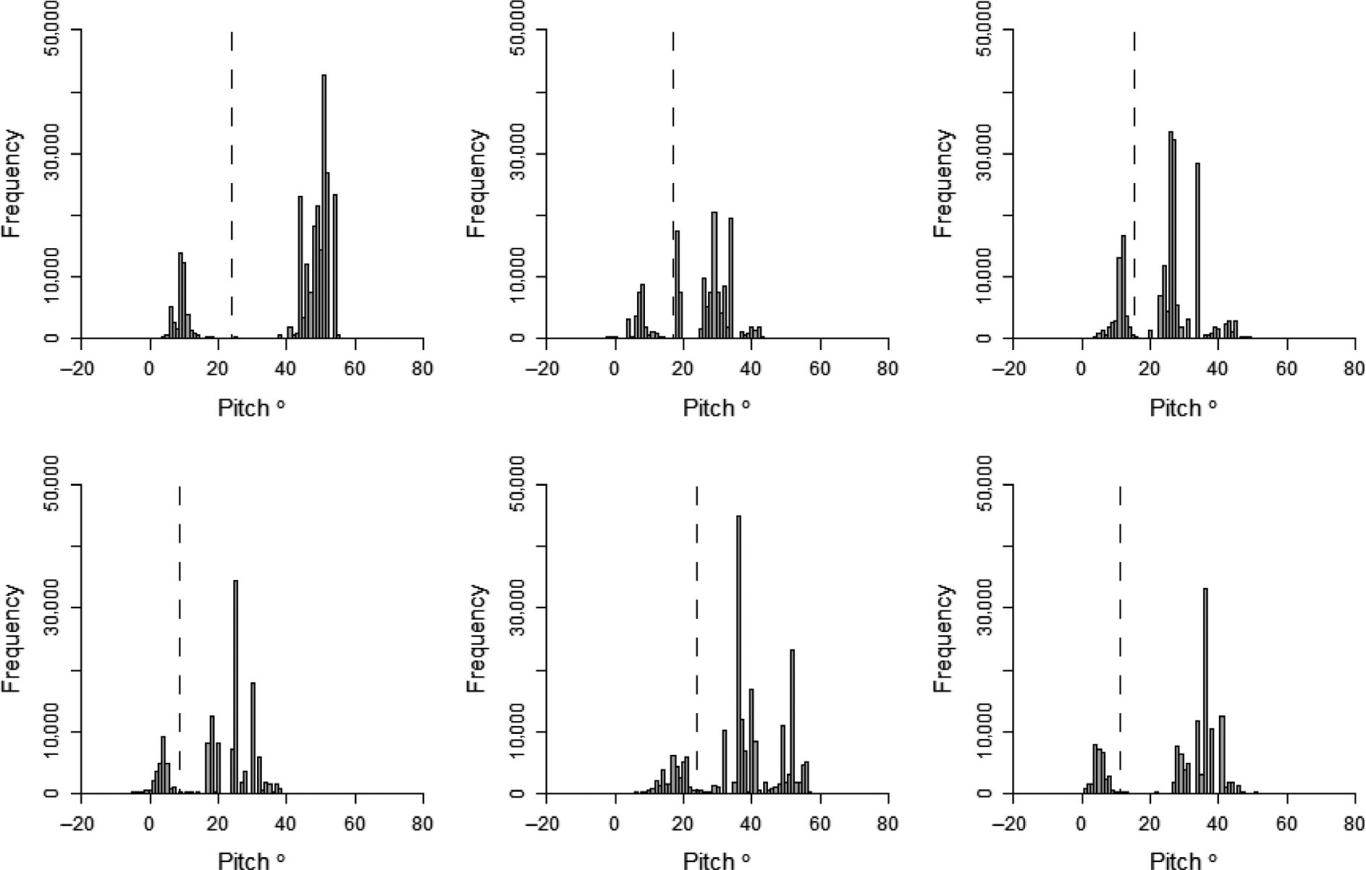
Histograms of pitch for each kittiwake after averaging pitch values between flight periods. The dashed line indicates the interpeak frequency minimum between the first and second peak present in the histogram before averaging (Fig. 6).

**Figure 8:**
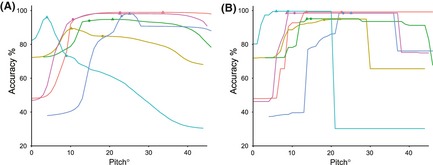
(A) Percentage accuracy of behavior assignments after determining whether the bird was on land or on water against body pitch. (B) Percentage accuracy of behavior assignments against body pitch after pitch values were averaged between bouts of flight. Circles indicate accuracy at the threshold value corresponding to the interpeak frequency minimum between the first and second peak from the histogram of pitch for each bird, while triangles indicate the value corresponding to the threshold value that achieves maximum accuracy.

Histograms for body pitch of the bird did not display such a clear or consistent distribution as histograms for SD_Heave_ (Fig. 6). Three of the birds had a distribution with two peaks in frequency, whereas the other three had three peaks. The degree to which these peaks were distinct, and at which point they occurred in the data varied between the individuals. However, averaging pitch values between flight periods further separated the peaks (Fig. 7) and, despite the variability between individuals, separating the behaviors of “on land” and “on water” by pitch was consistently highly accurate. By separating these behaviors using the threshold corresponding to the interpeak frequency minimum value between the first and second peak of each pitch histogram, accuracy of assignment was 90.4 ± 8.9% when behaviors were assigned based on initial pitch values, and 97.5 ± 2.1% when assigning behaviors based on the pitch values averaged between bouts of flight. The maximum possible accuracy of assignment by separating these behaviors by pitch was 95.9 ± 3.6% when assigned by initial pitch values and 97.7 ± 2.0% when pitch was averaged (Fig. 8A and B). In addition, the range of pitch values at which accuracy of assignment remained above 95% increased by an average of 8.5° ± 6.0° after assigning behaviors based on average pitch between bouts of flight. This is shown by the elongated plateaus of higher accuracy values in Figure 8B compared to Figure 8A.

### Humans

**Figure 9:**
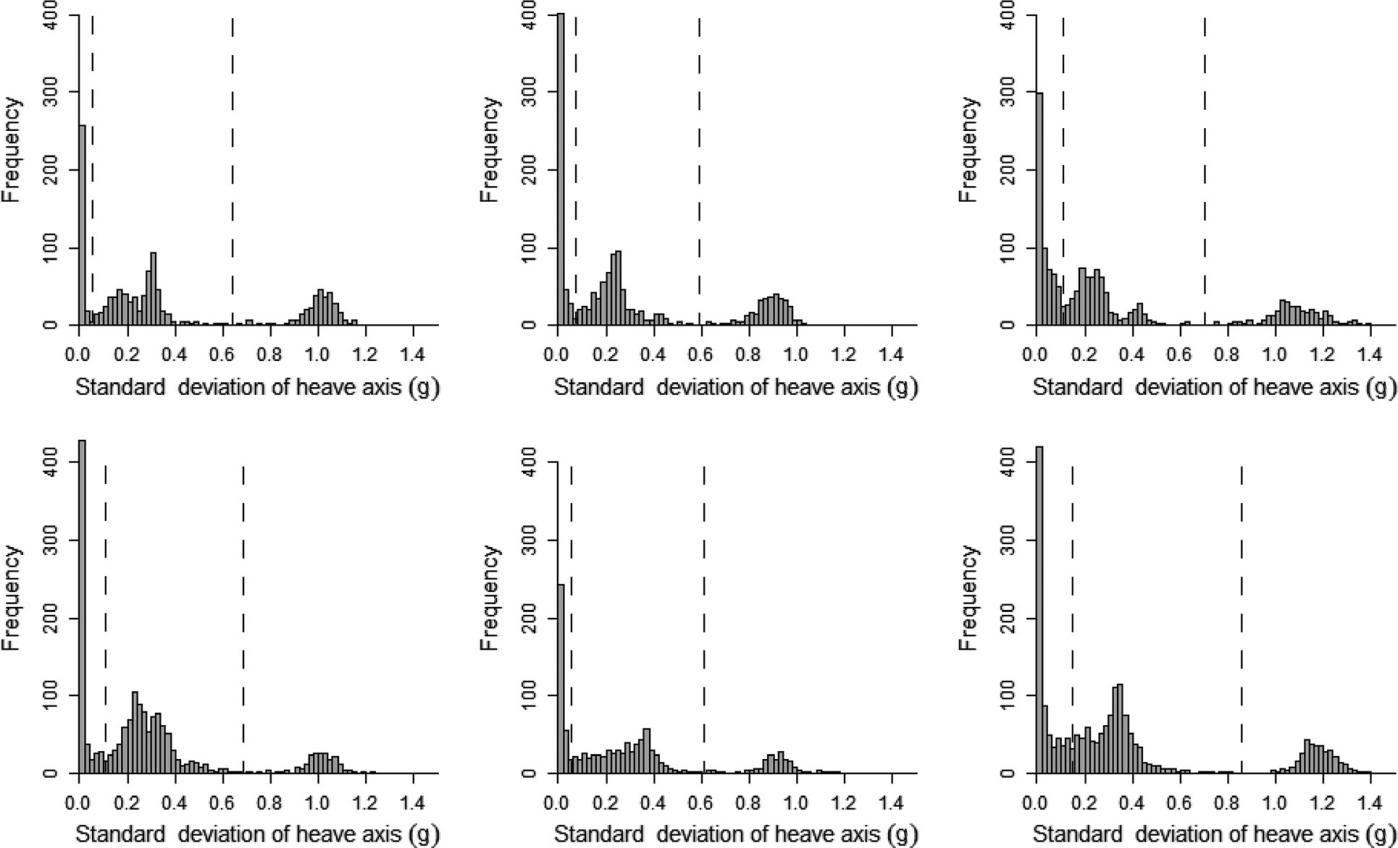
Histograms of the standard deviation of the heave axis data recorded during acceleration data logger deployments on six human participants. Dashed lines indicate the interpeak frequency minimum between peaks.

**Figure 10:**
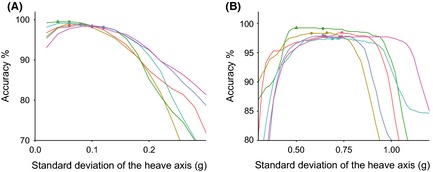
Percentage accuracy for all human participants against the standard deviation of the heave axis for (A) identifying sitting and (B) for separating walking and running behaviors. Circles indicate accuracy at the threshold value corresponding to the interpeak frequency minimum between (A) the first and second and (B) the second and third peak from the histogram of standard deviation of the heave axis for each participant. Triangles indicate the value corresponding to the threshold value that achieves maximum accuracy.

A trimodal distribution was present in histograms of SD_Heave_ for all human participants (Fig. 9). In this instance, SD_Heave_ was used to differentiate between all three behaviors exhibited (sitting, walking, and running). Separating sitting behavior from any movement using the interpeak frequency minimum of the first and second peak, assignment accuracy was 98.75 ± 0.68% (Fig. 10A). The highest possible percentage accuracy was higher than this at 99.11 ± 0.46%; the mean difference in accuracy was therefore small, at 0.36 ± 0.30%. Running and walking behaviors were separated after sitting data were already assigned. Using the interpeak frequency minimum value between the second and third peak of the standard deviation histogram to determine the threshold value, average assignment accuracy was 98.26 ± 0.88% (Fig. 10B). The highest possible accuracy regardless of frequency of standard deviation values was 98.42 ± 0.86%.

## Discussion

The analysis presented shows that by assigning behaviors using objectively determined thresholds from histograms of readily calculated metrics of accelerometry data, it is possible to classify coarse‐scale behaviors in both kittiwakes and humans to a high degree of accuracy. Estimated percentage accuracy of assignments of approximately 97% for kittiwake data and 98% for human data is very high, and such accuracy is comparable to methodologies achieving the highest rates of coarse‐scale behavior assignment (Nathan et al. 2012; Bidder et al. 2014; McClune et al. 2014). It should, however, be noted that a direct comparison to other methods has not been made.

The purpose of this study was to test and provide a method for assigning behaviors which can be readily applied to other datasets. Existing studies have used similar threshold based approaches to classify behavior (Yoda et al. 2001; Gómez Laich et al. 2009; Nathan et al. 2012; Kawabata et al. 2014). However, the threshold values provided in these cases have tended to be study specific, with little information given as to how such values were determined. In the present study, it has been demonstrated that separation of coarse‐scale behaviors can be achieved by assigning behaviors based on an objectively identified threshold value between peaks within histograms of suitable metrics of acceleration. By defining these thresholds as the value corresponding to the minimum frequency of data points falling between peaks (the interpeak frequency minimum), accuracy was almost as high as the maximum possible accuracy calculated for separating behaviors. As determining the interpeak frequency minimum is an objective stage of the method, the small difference in accuracy achieved when compared to the maximum possible accuracy achievable through an iterative approach of testing a range of threshold values justifies the application of this approach. This is especially true for studies where validation is not possible. Using objectively determined thresholds for separating behaviors is also advantageous in that they are specific to each individual while being simple to calculate. This reduces potential assignment error of using one threshold for all individuals which may arise from individual variation in the metrics used to separate behaviors. Furthermore, demonstration of the consistency of this approach for two distinct model species with contrasting behavioral modes implies that the method is likely suitable for a range of other species. In addition, unlike with more complex approaches incorporating machine learning for classifying behavior, which represent and classify data as points in space based on summary statistics (Bidder et al. 2014), the method outlined here relies on assigning behaviors based on metrics relating to the position of the subject (body pitch) or its amount of movement (standard deviation of an axis). This aspect of the method does incorporate some subjectivity into the method, at the point of choosing how many behaviors to classify and which metrics to use, but results in the process of assignment being readily understandable and justifiable in relation to the target species’ biology. With such metrics relating to behavior in many taxa, and the method being simple to execute, application of this approach on other species should be straightforward. Indeed, by providing the script to apply this method, we hope it will be further tested on acceleration data from species with different modes of behavior to those presented here.

In some cases, it may be that the shapes of histograms of chosen metrics do not correspond clearly with the number of behaviors being assigned. This was evident when using body pitch to separate the behaviors of “on land” and “on water” for kittiwakes, which was initially the least accurate stage of behavioral assignment. This was due to the pitch of the bird sometimes overlapping when on land and on water. Such overlap of pitch is likely to be due to the potentially small difference in orientation of the birds when on the nest in relation to their position on water. Pitch measurements were also likely to vary due to individual variation in amount of movement when on land (i.e., when the bird was mainly on the nest). However, the simplicity of the metrics used to separate these behaviors allowed for the inclusion of a biological argument to further enhance accuracy of assignments, namely that to transition between being on land and on water requires a period of flight between the two. Averaging pitch between bouts of flight further separated out the range of pitch values associated with the bird being on land and the range of values associated with the bird being on water, thus increasing accuracy.

Although pitch has been used to differentiate behavior in seabirds before (Shepard et al. 2008; Gómez Laich et al. 2009), species used in such studies have tended to have a much more defined difference in body angle between behaviors; for example, penguins and shags, which tend to be in either prone or upright positions during particular behaviors (Yoda and Ropert‐Coudert 2007; Gómez Laich et al. 2009). By averaging pitch between flight periods, this method can potentially be applied to other species which either overlap in pitch between behaviors or have less pronounced differences between body orientations across different behaviors. In addition to, and perhaps more important than, the increase in accuracy resulting from averaging pitch between flight bouts, the range of pitch values at which accuracy remained high increased in all birds. This effectively reduces the importance of identifying an exact threshold value for separating behaviors as long as the value identified falls in the range corresponding to high accuracy of assignment. While it is unlikely that such an argument can be applied to all taxa, where possible the inclusion of such biologically grounded arguments should be considered before resorting to more complex approaches of behavioral classification.

An unexpected consequence of our approach is that variation in frequency histograms of metrics such as body pitch could also be used as a diagnostic tool for identifying even coarser scale behavioral or life‐history states such as the stage of the breeding cycle of a target individual. The kittiwake individuals in this study which displayed three peaks in the pitch histograms were all rearing chicks while those with two peaks were incubating eggs. This is consistent with incubating birds spending a larger proportion of their time sitting (incubating), whereas chick rearing birds switch between sitting (brooding) and standing. This potential application of acceleration metric histograms could be especially viable given the continuing miniaturization and increased longevity of data logging devices (Hunt and Wilson 2012), which should enable longer term deployments on free‐ranging animals.

### Validation of behavior assignments

Validation of behavioral assignments on wild animals is often unobtainable. However, the approach of simultaneous deployment of two different types of logger, as demonstrated with coupling accelerometers with salt water immersion loggers on kittiwakes in this study, offered a source of sample validation. Such coupling of devices increases the confidence of interpreting information from datasets which may otherwise be difficult to justify (Wilson et al. 2008; Dean et al. 2013; Watanabe and Takahashi 2013). Furthermore, by allowing estimation of accuracy across a range of threshold values, this approach has enabled confirmation that frequency distributions (represented by histograms) of metrics of accelerometry data can indeed correspond to distinct behaviors. Although validation of behavioral assignments would be desirable for each study employing the accelerometry technique, it is not always possible. Using data from similar species, or even captive animals, to inform behavioral assignments (Campbell et al. 2013) has been suggested in the absence of validation; however, the approach we present here offers a solution which is not reliant on a source of validation, or sourcing other datasets. The lack of dependence upon validation therefore broadens the applicability of this approach.

## Conclusion

There are numerous methodologies available for classification of behavior from accelerometry data, for example (Shepard et al. 2008; Nathan et al. 2012; Brown et al. 2013). The present approach offers a method informed by sound biological reasoning for classifying coarse‐scale behaviors by means of objectively determined threshold values, and which is easy to understand, visualize and undertake. In turn, we hope that future studies of animal behavior based on the deployment of acceleration data loggers can employ the methods described here, thus bringing a degree of consistency to studies in which behaviors are assigned to accelerometry data. We especially hope for this method to be applied to and tested on a wider range of species exhibiting different types of behaviors. Where a more detailed behavioral analysis is required, the approach presented here offers an appropriate platform prior to further interrogation of the data. Such further analysis could, for example, involve isolating flight behavior to calculate wingbeat frequency or other such metrics now calculable from high‐resolution accelerometry data (Spivey and Bishop 2013).

## Conflict of Interest

None declared.

## Supporting information


**Appendix S1**. Histograms for calculated metrics of accelerometry from one kittiwake.
**Appendix S2**. Histograms for calculated metrics of accelerometry from one human participant.
**Appendix S3**. Histograms for calculated metrics of accelerometry from one kittiwake after data assigned as flight were removed.

**Appendix S4**. Accuracy of assignments for both species and all three behaviours depending on bin size used in the histograms generated to inform behavioural assignments.

**Appendix S5.** R Script for implementing the behavioral assignment method presented.

**Appendix S6.** An example accelerometry dataset from a kittiwake.
