# Supplementary material for: Interpreting behaviors from accelerometry: a method combining simplicity and objectivity
Source: Ecol Evol. 2015 Oct 2;5(20):4642–54. doi: 10.1002/ece3.1660 (PMC4670056; doi:10.1002/ece3.1660)

Appendix S1 –Histograms for calculated metrics of accelerometry from one kittiwake.

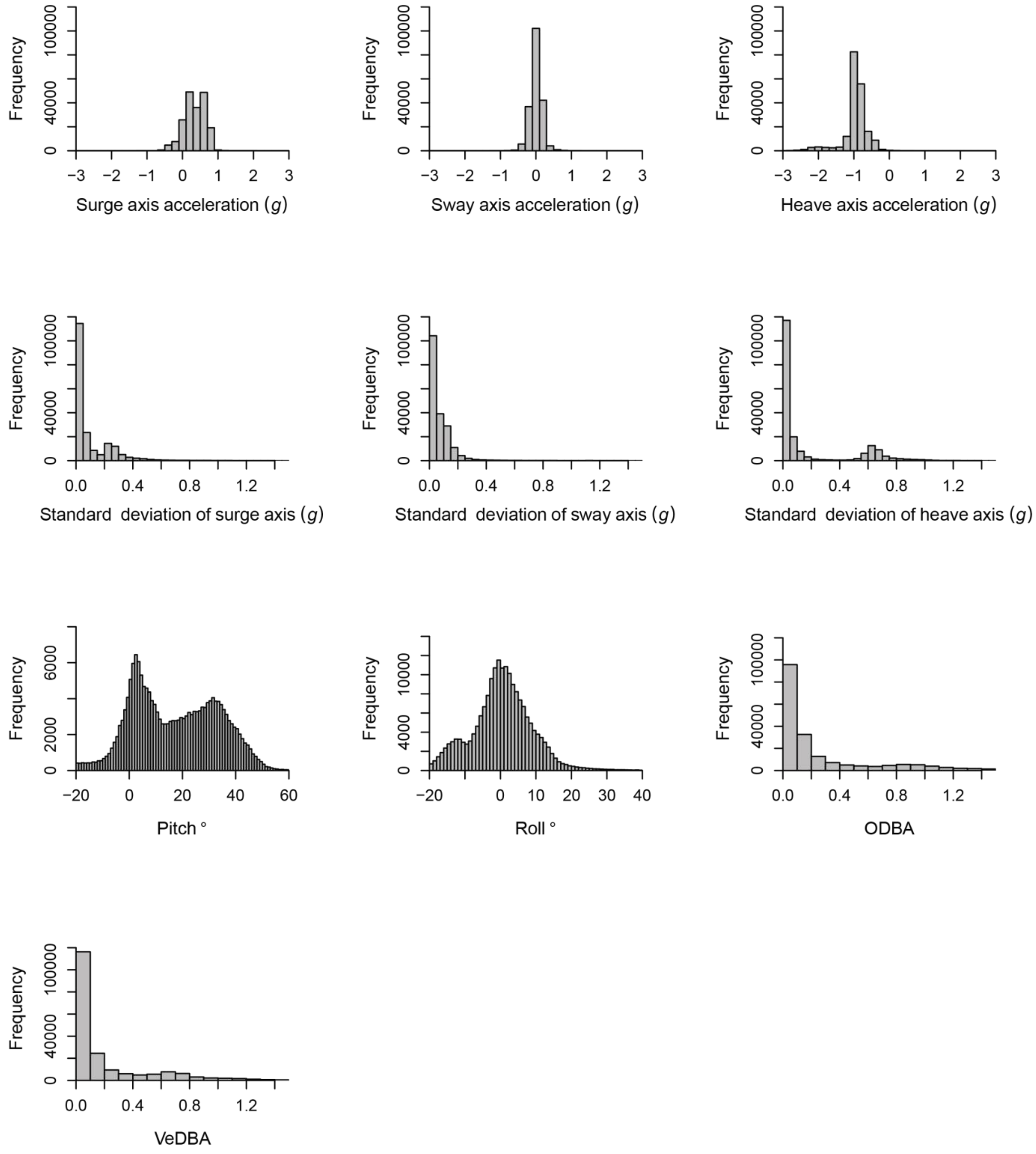

Appendix S2 –Histograms for calculated metrics of accelerometry from one human participant.

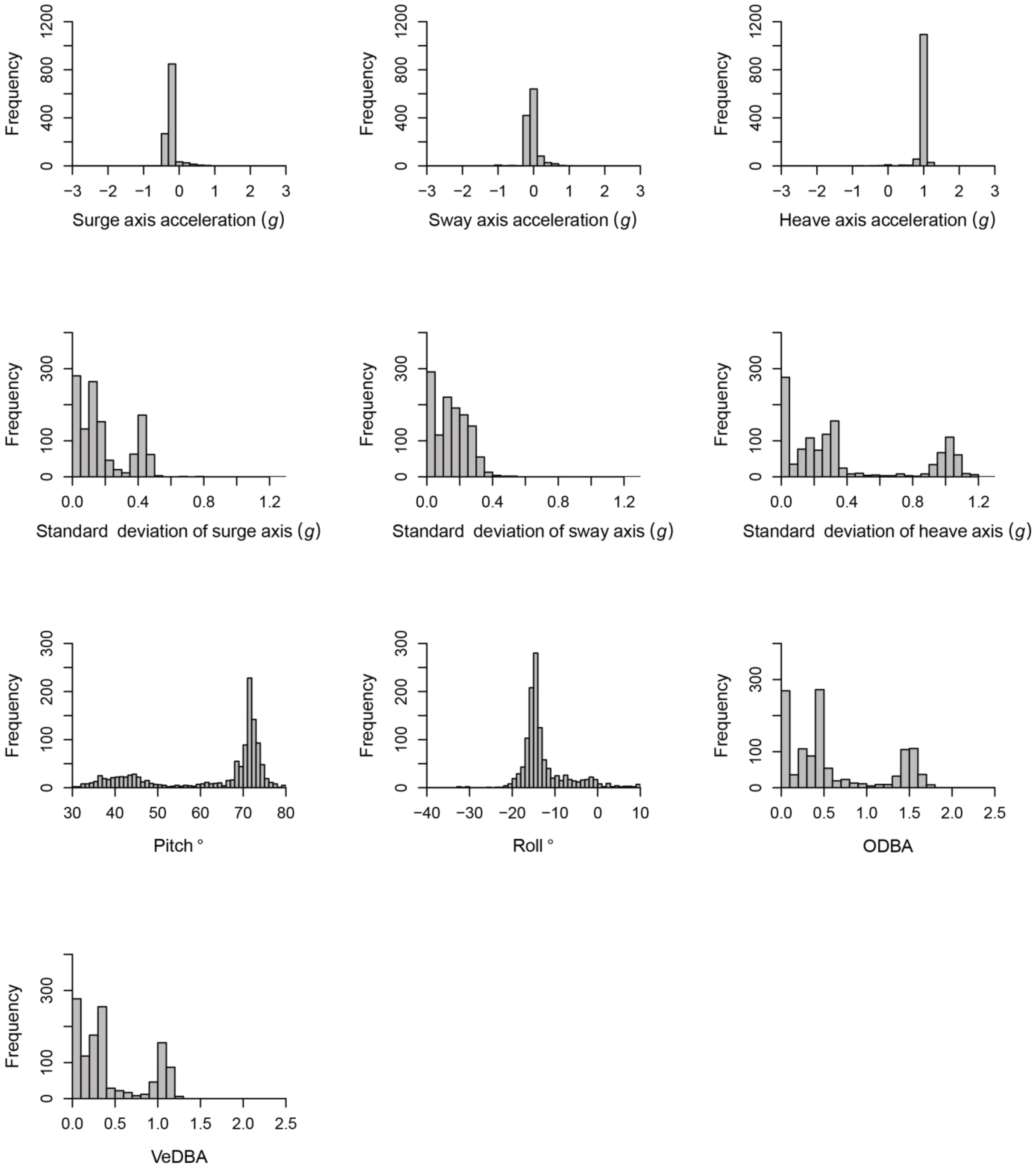

Appendix S3 –Histograms for calculated metrics of accelerometry from one kittiwake after data assigned as flight were removed.

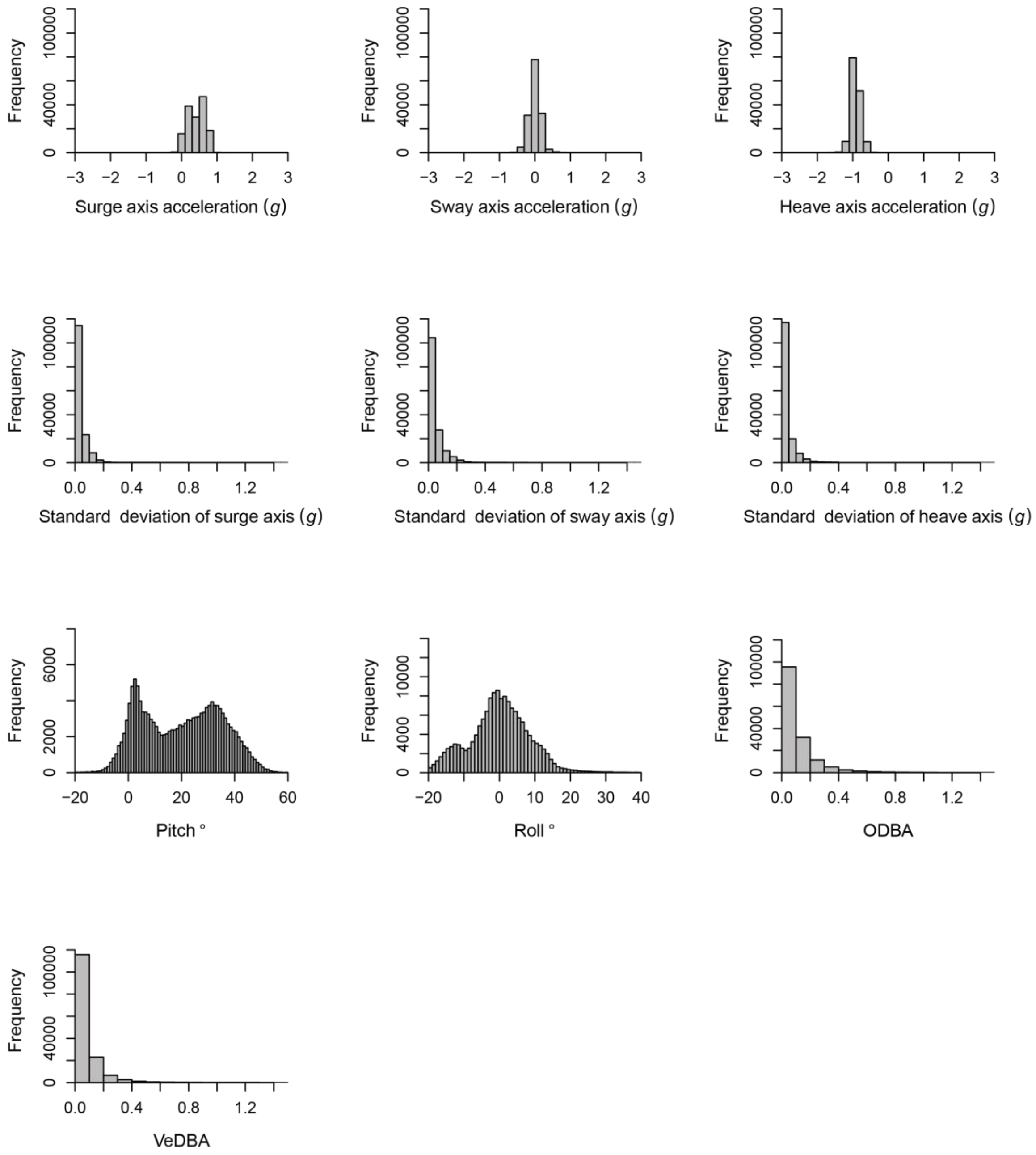

Supplement: Supplementary file 1 — Appendix S1. Histograms for calculated metrics of accelerometry from one kittiwake. Appendix S2. Histograms for calculated metrics of accelerometry from one human participant. Appendix S3. Histograms for calculated metrics of accelerometry from one kittiwake after data assigned as flight were removed. [file ECE3-5-4642-s001.pdf]
