# Supplementary material for: Interpreting behaviors from accelerometry: a method combining simplicity and objectivity
Source: Ecol Evol. 2015 Oct 2;5(20):4642–54. doi: 10.1002/ece3.1660 (PMC4670056; doi:10.1002/ece3.1660)
Supplement: Supplementary file 2 — Appendix S4. Accuracy of assignments for both species and all three behaviours depending on bin size used in the histograms generated to inform behavioural assignments. [file ECE3-5-4642-s002.docx]

**Appendix S4** –Accuracy of assignments for both species and all three behaviours depending on bin size used in the histograms generated to inform behavioural assignments.

|  | **Standard deviation of heave axis bin size** | | | | | | | |  | | **Pitch bin size** | | | | | |
| --- | --- | --- | --- | --- | --- | --- | --- | --- | --- | --- | --- | --- | --- | --- | --- | --- |
|  | | **0.01** | | **0.02** | **0.05** | **0.1** | **0.2** | **0.5** | |  | | **0.5** | **1** | **2** | **5** | **10** |
|  | | | % correct assignment | | | | | | |  | | % correct assignment | | | | |
| **Bird ID** | |  | |  |  |  |  |  | |  | |  |  |  |  |  |
| EG79073 | | 98.9 | | 98.9 | 98.7 | 98.8 | 98.3 | 98.1 | |  | | 98.8 | 98.8 | 98.9 | 98.9 | 98.9 |
| EL48867 | | 95.4 | | 95.7 | 95.0 | 96.0 | 95.3 | 96.4 | |  | | 93.8 | 93.8 | 93.7 | 93.8 | 94.3 |
| EL48898 | | 96.0 | | 96.1 | 96.0 | 95.8 | 95.2 | 96.2 | |  | | 95.5 | 95.5 | 95.8 | 95.8 | 94.5 |
| ET41812 | | 99.5 | | 99.5 | 99.5 | 99.4 | 99.5 | 99.5 | |  | | 99.5 | 99.5 | 99.5 | 99.5 | 99.5 |
| EX41203 | | 96.0 | | 96.0 | 96.1 | 96.2 | 96.1 | 97.0 | |  | | 95.8 | 95.8 | 96.5 | 96.6 | 74.6 |
| EX41446 | | 98.4 | | 98.4 | 98.4 | 98.5 | 98.4 | 98.7 | |  | | 98.4 | 98.4 | 98.4 | 98.4 | 75.2 |
|  | |  | |  |  |  |  |  | |  | |  |  |  |  |  |
|  | |  | |  |  |  |  |  | |  | |  |  |  |  |  |
| **Human ID** | |  | |  |  |  |  |  | |  | |  |  |  |  |  |
| 1 | | 94.9 | | 94.9 | 97.9 | 95.1 | 75.8 | 81.1 | |  | |  |  |  |  |  |
| 2 | | 98.4 | | 98.4 | 98.2 | 92.7 | 96.9 | 70.0 | |  | |  |  |  |  |  |
| 3 | | 97.5 | | 94.1 | 98.0 | 95.2 | 97.6 | 74.7 | |  | |  |  |  |  |  |
| 4 | | 97.6 | | 97.8 | 98.2 | 95.6 | 76.9 | 84.1 | |  | |  |  |  |  |  |
| 5 | | 99.4 | | 99.6 | 99.6 | 99.6 | 99.3 | 97.0 | |  | |  |  |  |  |  |
| 6 | | 96.0 | | 97.7 | 97.3 | 96.2 | 60.4 | 86.2 | |  | |  |  |  |  |  |
|  | |  | |  |  |  |  |  | |  | |  |  |  |  |  |
